# HPV-Associated Head and Neck Cancer: Molecular and Nano-Scale Markers for Prognosis and Therapeutic Stratification

**DOI:** 10.3390/s120405159

**Published:** 2012-04-20

**Authors:** Adam J. Kimple, Alexandra D. Torres, Robert Z. Yang, Randall J. Kimple

**Affiliations:** 1 School of Medicine, University of North Carolina, Chapel Hill, NC 27599, USA; E-Mail: kimplead@gmail.com; 2 Department of Human Oncology, University of Wisconsin, Madison, WI 53706, USA; E-Mails: alexandradtorres@gmail.com (A.D.T.); bobyang@wisc.edu (R.Z.Y.); 3 McArdle Laboratory for Cancer Research, University of Wisconsin, Madison, WI 53706, USA; 4 Carbone Cancer Center, University of Wisconsin, Madison, WI 53792, USA

**Keywords:** human papillomavirus, head and neck cancer, *in situ* hybridization, next generation sequencing, biomarkers

## Abstract

Over the last 10 years, it has become clear that patients with head and neck cancer can be stratified into two distinct subgroups on the basis of the etiology of their disease. Patients with human papillomavirus-related cancers have significantly better survival rates and may necessitate different therapeutic approaches than those with tobacco and/or alcohol related cancers. This review discusses the various biomarkers currently in use for identification of patients with HPV-positive cancers with a focus on the advantages and limitations of molecular and nano-scale markers.

## 1. Introduction

Squamous cell carcinoma of the head and neck is the sixth most common cancer worldwide, with nearly 50,000 cases diagnosed annually [1]. Patients are often diagnosed with locally advanced (*i.e.*, stage IV) disease with a significant burden of lymph node involvement. Optimal treatment for these patients usually involves evaluation in a multidisciplinary setting with the coordination of surgery, chemotherapy, and radiation therapy. In many cases combinations of therapy are used (reviewed in [2]).

Over the last 10 years, a growing proportion of patients with head and neck cancer have been found to have tumors attributable to the human papillomavirus (HPV). This virus has long been known to cause cancers of the uterine cervix in women and of the anal canal in both men and women. Initially described in head and neck cancer by Syrjanen and colleagues due to histologic similarities between oropharyngeal cancers and cervical cancers [3], HPV is now thought to cause 30–65% of head and neck cancers [4,5].

## 2. Importance of Human Papillomavirus in Head and Neck Cancer

There are currently more than 100 HPV subtypes that can be classified into low-risk and high-risk on the basis of their association with invasive malignancies. Just over a dozen HPV types have been classified as high risk. Of these, head and neck cancer is almost exclusively caused by HPV-16 which accounts for >90% of cases [6,7]. This is in stark contrast to cervical cancer where HPV-16 and HPV-18 together account for only approximately 70% of cases [8,9]. To date, it is not known why head and neck cancers arise almost exclusively as a result of HPV-16 infection although it has been postulated to be due to localization of a factor (not yet identified) necessary for HPV internalization on the epithelial surface of the head and neck.

In addition, HPV does not appear to affect all mucosal head and neck sites equally [6]. The vast majority of HPV-positive head and neck cancers arise from the mucosa lining the oropharynx (*i.e.*, tonsil, base of tongue, and soft palate) where the rate of HPV positivity has been as high as 80% in some studies [6,10,11]. Given the proximity of head and neck subsites (e.g., tonsil *vs.* retromolar trigone) and the difficulty in assigning a site of origin in some large primary tumors, it is also possible that HPV-positive cancers arising in other head and neck subsites are actually misclassified and truly arise from either the base of tongue or the tonsil.

There appears to be significant geographic and temporal variation in the rates of HPV-positive head and neck cancers with higher rates in the US compared to Europe (47% *vs.* 28%, respectively) [12] and several studies showing an increasing incidence over the last 20 years [13,14]. Whereas the rates of HPV-negative HNSCC and incidence of oral cavity tumors have seen a slight decrease over the same period of time [7,15].

HPVs are DNA viruses that are encoded by approximately 8000 base pair genomes. The double stranded circular DNA encodes eight proteins: E1, E2, E4, E5, E6, E7, L1, and L2. Carcinogenesis is thought to be driven by expression of E5, E6 and E7 with the other early genes playing important roles in viral gene transcription and viral DNA replication. L1 and L2 encode the capsid proteins which form the “coat” of the virus and which are targeted by HPV-vaccines (a topic beyond the scope of this review).

Interestingly, while patients with HPV-associated head and neck cancers commonly present with more advanced disease, they have significantly improved outcomes compared with stage and comorbidity matched HPV-negative patients. Differences in five year overall survival between HPV-positive and HPV-negative patients exceed 30% in a number of retrospective analyses [10,16–19]. This difference is one of the largest yet identified for cancers that arise within the same tissues, have very similar patterns of spread, and have overlapping histology. Interestingly, even within patients with HPV-positive HNSCC, those with a history of significant tobacco/alcohol use show significantly worse outcomes than never smokers; but an outcome that remains better than those with HPV-negative disease [5].

These large differences in outcome have arisen in an era during which patients with HPV-positive cancers were treated no differently from those with HPV-negative cancers. However, in the past several years the oncology community has begun to think about HPV-positive head and neck cancer as a different disease than traditional tobacco/alcohol related head and neck cancer [20–22]. It is hoped that HPV-status may ultimately aid in selecting treatment options. However, due in part to difficulties in determining whether a given patient's tumor is HPV-positive or HPV-negative, clinical trials specific for HPV-positive patients have only recently begun enrolling patients (e.g., NCT01302834, NCT01530997, NCT01525927, NCT01221753, NCT01084083). In this article we will review the current state-of-the-art regarding biomarkers to identify patients with HPV-positive cancers with a focus on the advantages and limitations of molecular and nano-scale markers.

## 3. Non-Amplified Detection

### 3.1. Southern Blot: The Gold Standard

**Figure 1.**
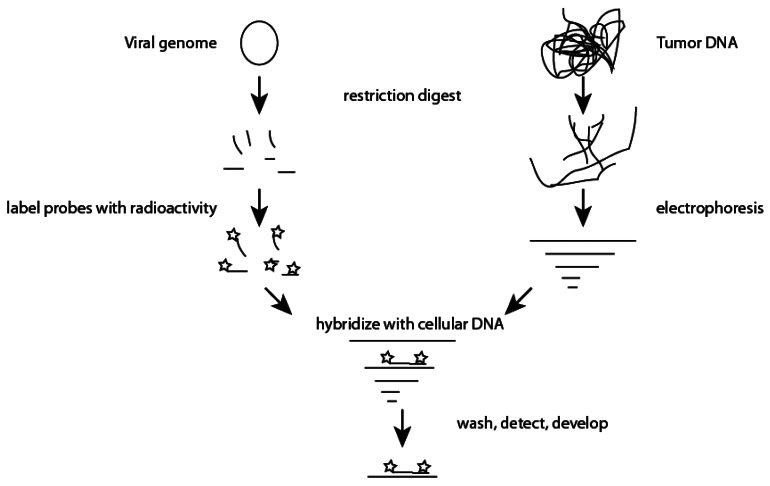
A type specific viral genome is digested with chosen restriction enzymes and resulting oligonucleotide fragments are radioactively labeled. Simultaneously, tumor DNA is also subjected to restriction digestion. Tumor DNA fragments are separated by agarose electrophoresis and transferred to a nitrocellulose membrane. Radioactively labeled probes are allowed to hybridize with cellular DNA, washed, and detected via overnight exposure of film to the membrane. Bands of HPV DNA present within the original tumor DNA can be detected due to hybridization with labeled probes.

Originally described by Edwin Southern in 1975, the “Southern Blot” is the gold standard test to measure the number of copies of a given gene or to analyze stretches of DNA that are too repetitive for PCR amplification or classical sequencing methods [23,24]. While techniques very considerably, Southern blots are labor intensive pursuits that require isolation of relatively large amounts of genomic DNA, digestion with restriction endonucleases, separation of DNA by electrophoresis, transfer of DNA to a nitrocellulose membrane, synthesis of radio-labeled nucleic acid probe(s), hybridization, and finally, exposure of film (Figure 1).

At this time, these steps are not easily amenable to automation. In addition, and of concern to a clinical lab, they typically involve multiple wash steps that produce large volumes of dilute radioactive waste. While Southern blots are labor intensive, they have an important role in studying tumor viruses such as HPV as they allow one to detect integration of the viral genome into the host genome. Additionally, assays can be developed with wash parameters and probes that are well suited to screening for multiple HPV subtypes. Southern blots can also be used to confirm the presence of specific HPV subtypes [25]. When compared to PCR based methods, Southern blot techniques have much lower false positive rates due to the detection of both a DNA fragment and the specific size of a digested DNA fragment. Similar to PCR based methods; Southern blots can be optimized to detect <0.1 copy of a given DNA sequence per cell giving them high sensitivity. However, in contrast to PCR based strategies, which inherently amplify the starting material, each step in a Southern blot results in loss of a proportion of the starting DNA. Thus the quantity of DNA needed for a Southern blot is orders of magnitude higher than that required for PCR based strategies.

### 3.2. *In Situ* Hybridization

**Figure 2.**
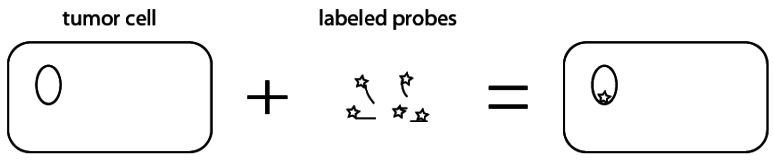
Tumor blocks are unmasked and allowed to hybridize with HPV type specific probes prior to fixation and detection via either fluorescent or bright field microscopy. HPV DNA sequences within the nucleus of cells can be identified.

*In situ* hybridization (ISH) is a commonly used in the diagnostic lab setting to test for HPV [6]. Using HPV type specific probes, ISH can detect either a single HPV subtype or a panel of high or low risk genomes [26]. HPV DNA within formalin-fixed and paraffin-embedded sections is targeted by using biotinylated HPV-specific probes [6]. This enables each probe to be detected using either colorimetric or fluorescent labels (*i.e.*, fluorescent ISH or FISH) that can be visualized using a standard pathology microscope (Figure 2). Signals originating within the nuclei of cells are usually associated with HPV genome integration, although this is not strictly true [6].

Compared to Southern blot hybridization, ISH has lower specificity for HPV detection [27] and lower sensitivity than amplification based methods. Important advantages of ISH include the ability to perform assays on formalin-fixed paraffin-embedded sections without significant additional processing, and the ability to visualize results on a standard microscope. In addition, one can determine whether the hybridization occurs in tumor tissue or in normal epithelial tissues.

### 3.3. Amplified Detection

#### 3.3.1. Polymerase Chain Reaction

**Figure 3.**
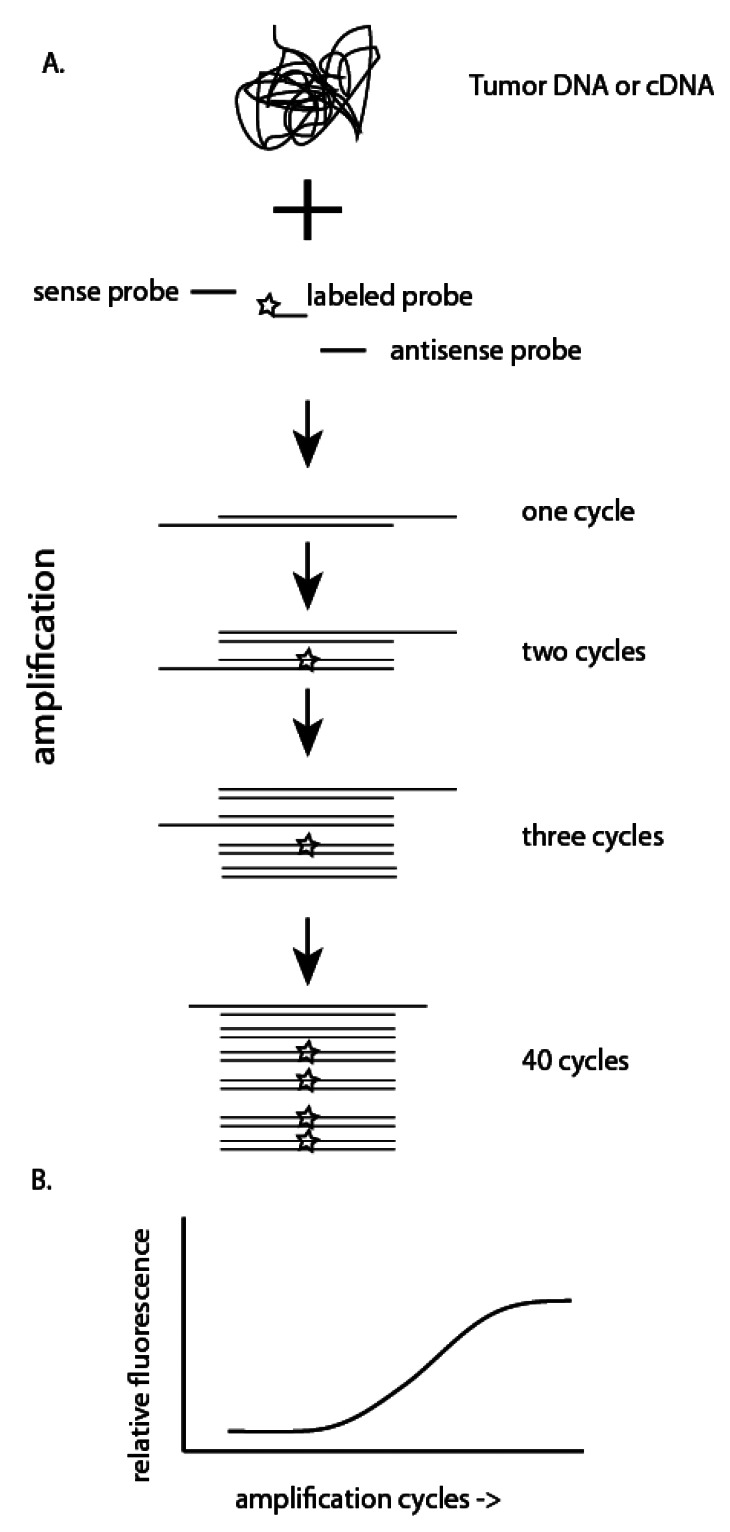
(**A**) Polymerase chain reaction (PCR) or reverse transcriptase PCR utilize either tumor DNA or cDNA that is reverse transcribed from tumor RNA. Oligonucleotide probes specific for a region of DNA are then used to amplify a given sequence region. The use of a labeled probe (star) allows for real-time detection of amplification products (**B**).

Polymerase chain reaction (PCR) allows for amplification of DNA isolated from tumor cells. Briefly, a DNA polymerase recognizes an oligonucleotide primer bound to a specific DNA sequence (Figure 3). By using two primers that flank a targeted region of interest, after several round of amplification, the target has been amplified sufficiently to allow for visualization on an agarose gel. Either degenerate primers which amplify DNA sequences from multiple subtypes of HPV, or specific primers which amplify DNA sequences from a single subtype of HPV can be used so that PCR can be used both as a screening test for any HPV infection and to confirm the subtype of HPV identified.

The resulting PCR products from one or more HPV subtype can be detected by oligonucleotide array. Briefly, type-specific probes are plated onto an array. The PCR product is hybridized to the chip and resulting signals are visualized with a DNA chip scanner. This type of assay can have a sensitivity approached 95% and has the added benefit of being able to detect multiple HPV types within a single specimen [28,29]. This type of assay can also be used to detect amplified mRNA sequences (discussed more below) that may correlate with progression to invasive disease [30].

Unlike standard PCR that amplifies genomic DNA, reverse transcriptase PCR (RT-PCR) utilizes RNA that is first reverse transcribed into cDNA. Following the generation of cDNA, PCR is performed as described above.

Quantitation of products for both standard PCR and RT-PCR can be performed by agarose gel electrophoresis. Alternatively, products can be detected in real-time with the use of DNA sequence-specific probes or fluorescent dyes (Figure 3). qRT-PCR allows for relative quantitation of RNA levels if appropriate controls are performed and can be used to determine whether an HPV infection is transcriptionally active (*i.e.*, does the viral DNA present result in production of mRNA and viral proteins). Due to the high dynamic range of qRT-PCR (>7 logs of input), one can detect RNA sequences present at very low concentrations that may not be identified by conventional PCR [31].

Both PCR and RT-PCR are highly sensitive tests owing to the exponential amplification of target sequences that lie between two priming sequences. In theory these techniques can detect a single copy of a target sequence within a given sample. In reality, this high sensitivity can lead to false positive results either through the inclusion of random HPV genomes (particularly troublesome in labs which commonly study HPV) or by detection of HPV genomes within the investigated tissues, but that are not causative for the malignancy. Probe based qRT-PCR, which utilizes a third probe that lies within the amplified region can significantly decrease the risk of false positives.

These tests can also be used to estimate the integration status of the HPV genome. Upon integration of the viral genome, both the L1 and E2 genes are typically disrupted and lost. Samples that retain E6 and E7 expression, but do not express L1 or E2 are considered to harbor integrated HPV [32,33] while those that express all 4 viral genes harbor episomal HPV. qRT-PCR methods can be automated at each step from purification of cellular RNA to production of cDNA to amplification, detection, and analysis so that little user effort is required. However, for both tests, the possibility of target degradation when fresh frozen tissue is unavailable represents a significant limitation in most clinical settings.

#### 3.3.2. Next-Generation Sequencing

**Figure 4.**
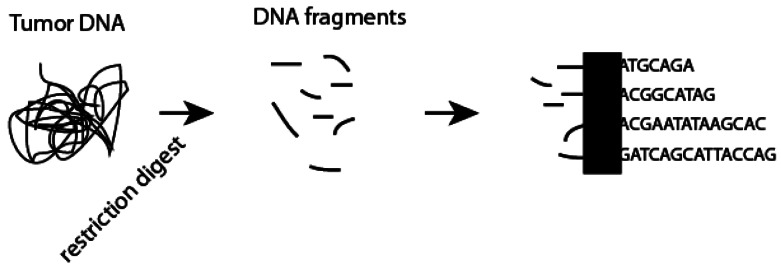
Next generation sequencing utilizes high-throughput methods to simultaneously determine the oligonucleotide sequence of hundreds or thousands of DNA fragments generated following restriction digestion of tumor DNA or reverse transcription of tumor RNA. Newer methods are also able to sequence RNA directly. Each commercial platform utilizes a different specific technology (black box) to determine the nucleotide sequence.

Next-generation sequencing (NGS) is a promising method for assessment of HPV-status on a nano scale. NGS utilizes high-throughput sequencing technology to simultaneously sequence many thousands of DNA or expressed RNA fragments (Figure 4). A number of different technologies are available (reviewed in [34] and [35]), but all determine the nucleotide sequences of DNA fragments simultaneously using ever-faster and more cost effective techniques. Over the last ten years, significant advances in NGS have been made so that it is now feasible to perform whole genome sequencing of an individual patient's tumor in a matter of hours.

Several groups have recently published proof-of-concept studies using two of the available next-generation technologies to assess for HPV status in cytology samples [36] and formalin fixed paraffin embedded samples [37]. NGS requires little input DNA making it an assay with extremely high sensitivity. In addition, to its ability to rapidly examine tumor samples for HPV DNA, NGS is able to determine both viral genome copy number and viral genome subtype. Finally, unlike other methods described above, NGS may identify co-infections with multiple HPV subtypes or associations with alternative viral etiologies.

### 3.4. Detection of Surrogate Markers

#### 3.4.1. HPV 16 Seropositivity

While not testing whether a given patient's tumor is related to HPV infection, exposure to HPV can be determined through serology. Using serum antibody detection systems, monoclonal antibodies raised against HPV type-specific epitopes are used to compete with a patient's antibodies produced in response to HPV infection or HPV immunization. Due to high background rates in the general population, HPV serology is not currently useful as a screening tool [38] and at the current time plays no role in managing patients with head and neck cancer [39].

#### 3.4.2. p16 Immunohistochemistry

Cyclin-dependent kinase inhibitor 2A (a.k.a., p16^Ink4A^ or p16) is a protein involved in cell cycle regulation. Expression of p16 has been shown to correlate with significantly improved outcomes in patients with head and neck squamous cell carcinomas [11,40]. This strong correlation has led to the suggestion that p16 expression be incorporated into staging guidelines for head and neck cancer [20]. Overexpression of p16 in HPV-positive cancers results from inactivation of Rb by the HPV protein E7 [41]. However, we and others have shown that not all p16 positive cancers are due to HPV infection [40,42].

Currently, whether patients with p16 positive, but HPV-negative cancers derive the same benefit from a given treatment as those with p16 positive, HPV-positive cancers remains unknown. In many centers, p16 testing is currently used as a surrogate for HPV status without actually detecting HPV DNA. To overcome this limitation, it has been proposed to couple p16 IHC with a secondary assay to directly detect HPV DNA or RNA [43].

In comparison to southern blot hybridization, *in situ* hybridization, PCR, or RT-PCR, the detection of p16 by IHC requires no specialized equipment or tissue handling. Nearly all pathology laboratories are well equipped for IHC and proprietary kits are commercially available for p16 staining in formalin-fixed, paraffin-embedded tissue [44]. Shi and colleagues recently performed both qRT-PCR and ISH for HPV-16 and found both to be associated with improved disease free survival in a Canadian cohort of patients with oropharyngeal squamous cell carcinoma [45]. In their study, concordance between HPV16 ISH and HPV-16 E6 mRNA specific qRT-PCR was 86% while IHC showed a 92% concordance with ISH, and an 86% concordance with E6 mRNA by qRT-PCR suggesting that any of these duplex methods of detection is a reasonable approach [45].

## 4. Conclusions

Our ability to detect HPV infection in head and neck cancer samples has greatly outpaced our ability to use this information to alter therapy. However, ongoing clinical trials and improved understanding of the molecular mechanisms underlying HPV-associated cancers promise to enable personalization of therapy on the basis of HPV-infection. At the current time limitations of alternative methods for detection of HPV result in the use of IHC for p16 as the de facto test for HPV-positivity (Table 1). However, as throughput increases and costs decrease, it appears likely that technologies that can accurately identify a particular HPV-subtype will gain traction.

## Figures and Tables

**Table 1.** Pros and cons for tests of HPV status.

**Test**	**Pros**	**Cons**
Southern blot	specific	sensitivity, requires large amounts of DNA, time intensive
ISH	specific, performed on FFPE specimens	poor sensitivity
PCR	specific, rapid	false positives
RT-PCR	sensitive and specific, rapid	requires intact RNA
NGS	sensitive and specific, detect multiple HPV types	cost
p16	perform in clinical labs, correlates with response	not specific for HPV
serology	easy to perform	No direct relationship to viral-associated cancer
